# The Effects of Endogenous Non-Peptide Molecule Isatin and Hydrogen Peroxide on Proteomic Profiling of Rat Brain Amyloid-β Binding Proteins: Relevance to Alzheimer’s Disease?

**DOI:** 10.3390/ijms16010476

**Published:** 2014-12-29

**Authors:** Alexei E. Medvedev, Olga A. Buneeva, Arthur T. Kopylov, Oksana V. Gnedenko, Marina V. Medvedeva, Sergey A. Kozin, Alexis S. Ivanov, Victor G. Zgoda, Alexander A. Makarov

**Affiliations:** 1 Department of Proteomic Research and Mass Spectrometry, Institute of Biomedical Chemistry, 10 Pogodinskaya Street, Moscow 119121, Russia; E-Mails: olbun@yandex.ru (O.A.B.); a.t.kopylov@gmail.com (A.T.K.); oksana_gnedenko@pochta.ru (O.V.G.); asi@icnet.ru (A.S.I.); vic@ibmh.msk.su (V.G.Z.); 2 Engelhardt Institute of Molecular Biology, Russian Academy of Sciences, Moscow 119991, Russia; E-Mails: kozinsa@gmail.com (S.A.K.); aamakarov@eimb.ru (A.A.M.); 3 School of Biology, Lomonosov Moscow State University, Moscow 119191, Russia; E-Mail: marmed64@yandex.ru

**Keywords:** amyloid-β, amyloid-β binding proteins, proteomic profiling, rat brain, isatin, oxidative stress

## Abstract

The amyloid-β peptide is considered as a key player in the development and progression of Alzheimer’s disease (AD). Although good evidence exists that amyloid-β accumulates inside cells, intracellular brain amyloid-binding proteins remain poorly characterized. Proteomic profiling of rat brain homogenates, performed in this study, resulted in identification of 89 individual intracellular amyloid-binding proteins, and approximately 25% of them were proteins that we had previously identified as specifically binding to isatin, an endogenous neuroprotector molecule. A significant proportion of the amyloid-binding proteins (more than 30%) are differentially expressed or altered/oxidatively modified in AD patients. Incubation of brain homogenates with 70 µM hydrogen peroxide significantly influenced the profile of amyloid-β binding proteins and 0.1 mM isatin decreased the number of identified amyloid-β binding proteins both in control and hydrogen peroxide treated brain homogenates. The effects of hydrogen peroxide and isatin have been confirmed in optical biosensor experiments with purified glyceraldehyde-3-phosphate dehydrogenase, one of the known crucial amyloid-β binding proteins (also identified in this study). Data obtained suggest that isatin protects crucial intracellular protein targets against amyloid binding, and possibly favors intracellular degradation of this protein via preventing formation of amyloid-β oligomers described in the literature for some isatin derivatives.

## 1. Introduction

The amyloid-β peptide **1**–**42** formed during proteolytic processing of the amyloid precursor protein (APP) is considered as a key player in the development or progression of Alzheimer’s disease (AD) and other pathologies associated with the formation of protein aggregates in the central nervous system ([1,2,3,4,5,6,7,8] and many others). Although much attention is paid to formation of extracellular amyloid plaques and protein aggregates as well as to corresponding mechanisms of their toxicity, good evidence exists that intracellular amyloid-β can accumulate intraneuronally and contribute to disease progression [4,9,10,11,12,13,14,15]. Results of experiments on transgenic mice indicate that the intraneuronal amyloid-β accumulation precedes plaque formation [16]. This suggests the importance of amyloid-β interaction with particular intracellular targets. Indeed, interaction of amyloid-β with intracellular catalase caused inactivation of this enzyme and accumulation of hydrogen peroxide inside cells [17]. This implies that oxidative stress induced in the cells exposed to amyloid-β may be (at least partially) associated with reduced degradation of intracellular hydrogen peroxide [17].

In the cell, amyloid-beta was found in different intracellular compartments [4]. The latter suggests the possibility of amyloid interaction with a broad range of proteins, which may be thus denominated as amyloid-β binding proteins. Although there are reports on the interaction of amyloid-β peptide with various intracellular proteins (e.g., [17,18]) and development of protocols for affinity isolation of amyloid-β binding proteins [19], the affinity based proteomic profiling of brain proteins interacting with immobilized amyloid-β has not been performed yet. Some previous studies revealed several important intracellular proteins involved in direct interaction with amyloid-β. These include glyceraldehydes-3-phosphate dehydrogenase (EC 1.2.1.12) (GAPDH) [20,21,22]. This glycolytic enzyme, a classical glycolytic redox sensitive enzyme, exhibits various non-glycolytic functions that are considered to be especially important for progression of various neurodegenerative diseases, particularly AD [23]. GAPDH is considered as a potential target for neuroprotective drugs. GAPDH interacts with isatin, an endogenous indole exhibiting properties of an endogenous neuroprotective molecule [24,25,26,27]. Previous studies have also shown that a significant proportion of rat (or mouse) brain proteins specifically interacted with isatin [28,29,30], are oxidatively modified in various AD models [11,31,32,33]. This suggests the existence of a possible interrelationship between capacities of at least some redox sensitive brain intracellular proteins to interact with both amyloid-beta and isatin.

Thus, in this study we have investigated proteomic profiles of beta-amyloid binding proteins of rat brain and their changes induced by hydrogen peroxide and/or isatin. The results of proteomic profiling have been validated in a surface plasmon resonance (SPR) based study with purified glyceraldehyde-3-phosphate dehydrogenase.

Data obtained suggest that interaction between amyloid-beta and its crucial intracellular targets may be influenced by (non-peptide) small organic molecules such as isatin and this opens new possibilities in pharmacological prevention of amyloid-beta toxicity.

## 2. Results

### 2.1. Proteomic Profiling of Amyloid-Binding Proteins of Intact Rat Brain Homogenate

**Figure 1:**
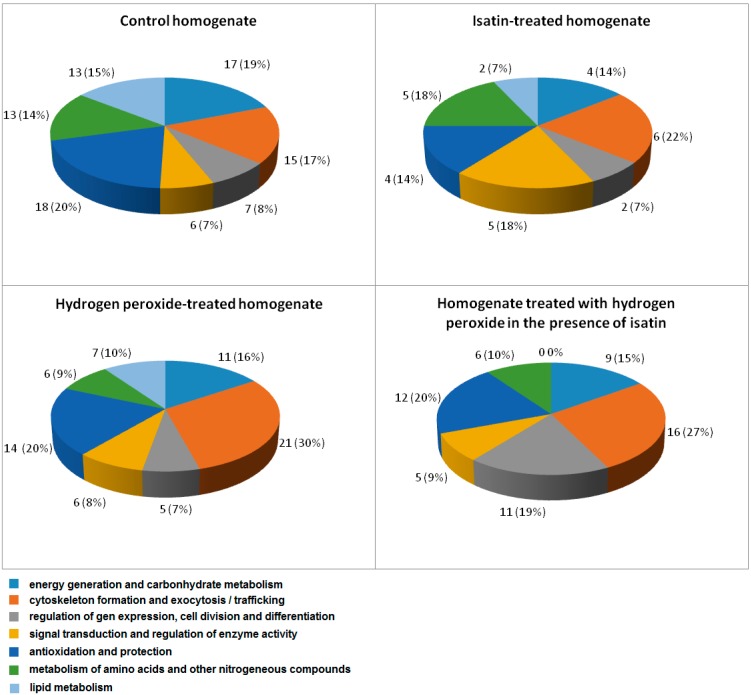
Subdivision of amyloid-binding proteins identified in brain homogenates by their functions: Numbers indicate total number of proteins in each group and numbers in parentheses show the percentage of the total number of identified proteins.

Loading of cleared Triton X-100 lysates of whole brain homogenate onto the affinity sorbent, amyloid-beta-Affi-Gel 10, followed by wash with 50 mM potassium phosphate buffer, pH 7.4, resulted in adsorption of 10.4% of the total protein applied. The control Affi-Gel 10 lacking the affinity ligand bound not more than 4% of the total protein applied. This suggests that about 6% of homogenate proteins specifically bound to the affinity sorbent. Subsequent elution of adsorbed proteins followed by their proteomic analysis resulted in identification of at least 89 individual intracellular β-amyloid binding proteins specifically bound to the affinity sorbent (Table S1). They have different intracellular localization and functionally, the identified proteins can be subdivided into the following groups (Figure 1): (i) Energy generation and carbohydrate metabolism; (ii) Cytoskeleton formation and exocytosis/trafficking; (iii) Regulation of gene expression, cell division and differentiation; (iv) Signal transduction and regulation of enzyme activity; (v) Antioxidant and protection proteins/enzymes; (vi) Metabolism of amino acids and other nitrogenous compounds; and (vii) Lipid metabolism. It is particularly significant that metabolic consequences of amyloid-beta interaction with some of these proteins (e.g., catalase, cytochrome oxidase) have already been documented and analyzed in the literature [17,18].

In accordance with our previous studies [28,29,30], among proteins non-specifically bound to the ligand-free control sorbent, we detected only highly abundant rat brain proteins [34]: The cytoskeletal keratins (10, 1, 6A, 14), and the mitochondrial precursor of β-subunit of ATP synthase. Interestingly, most of these proteins bound non-specifically to both Sepharose [29,30] and Affi-Gel. Thirty of eighty nine amyloid-binding proteins (more than 30%) were found to be oxidatively modified/altered either in brains of AD patients or in different experimental models of this disease (Table 1).

Twenty three of eighty nine amyloid-binding proteins (more than 25%) have been identified earlier [28,29,30] as proteins specifically bound to isatin (indole-2,3-dione), an endogenous indole which has a distinct and discontinuous distribution in the brain and in other mammalian tissues and body fluids [24,25,27]. Some of them undergo oxidative modification in AD (Table 1).

One of these oxidatively modified proteins which also interact with isatin is glyceraldehyde-3-phosphate dehydrogenase (GAPDH) [26].

**Table 1:** Amyloid-binding proteins altered or oxidatively modified in Alzheimer’s disease and related pathologies and their experimental models.

No.	Protein Name	Changes in Pathology or Experimental Model	Reference
1	Pyruvate kinase isozymes M1/M2	OM, I	[11,33,35]
2	Actin, cytoplasmic 1	OM, D	[36,37]
3	Heat shock cognate 71 kDa protein	OM	[38]
4	ATP synthase subunit β, mitochondrial	I	[39]
5	Alpha-enolase	OM, I, AALS	[33,35,40,41]
6	Myelin basic protein S	OM	[11]
7	Glutathione *S*-transferase α-2	OM	[11]
8	Dihydropyrimidinase-related protein 2	OM, I, D, AALS	[11,40,41,42]
9	60 kDa Heat shock protein, mitochondrial	D	[39]
10	Glyceraldehyde-3-phosphate dehydrogenase	OM	[11,33]
11	Fructose-bisphosphate aldolase A	OM	[33]
12	ATP synthase subunit α, mitochondrial	D	[39]
13	Endoplasmin	OM	[33]
14	Peroxiredoxin-1	I	[43]
15	Stress-70 protein, mitochondrial	I	[44,45,46]
16	Retinol-binding protein 4 OS	D	[47]
17	78 kDa Glucose-regulated protein	OM, AALS	[33,41]
18	Peroxiredoxin-2	OM, I	[33,43,48]
19	Glutamine synthetase	OM, I	[11,40]
20	Glutathione *S*-transferase P OS	OM	[11]
21	Glutamate dehydrogenase 1, mitochondrial	OM, AALS	[33,41]
22	Heat shock protein HSP 90-α	I	[49]
23	Spectrin α chain, non-erythrocytic 1	OM	[11]
24	Phosphoglucomutase-1	OM	[32]
25	Synaptotagmin-1	D	[44,50]
26	Phosphatidylethanolamine-binding protein 1	I	[51]
27	Calmodulin	D, AALS	[41,52]
28	Protein disulfide-isomerase	AALS	[41]
29	Superoxide dismutase [Mn]	OM	[53]
30	Tubulin α-1A chain	OM	[32]

I—Increased, D—Decreased, OM—Oxidatively modified, AALS—Altered affinity to lectin sorbents.

### 2.2. The Effects of Hydrogen Peroxide and Isatin on the Interaction of Glyceraldehyde-3-phosphate Dehydrogenase (GAPDH) with Immobilized Amyloid-β and Actin

**Figure 2:**
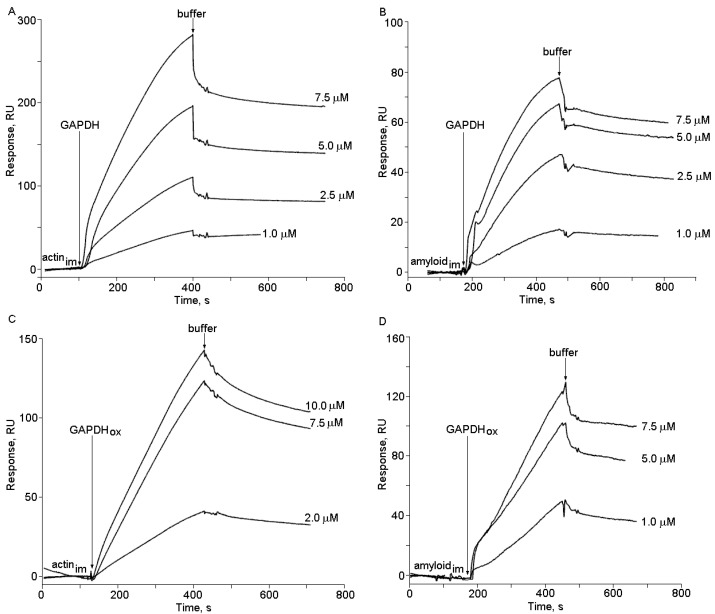
Sensograms of binding of native (**A**,**B**) and oxidized (**C**,**D**) glyceraldehyde-3-phosphate dehydrogenase (GAPDH) to immobilized actin (**A**,**C**) and amyloid-β (**B**,**D**). Arrows indicate onset of injection.

The injection of highly purified GAPDH to the biosensor flow cell with immobilized amyloid-β caused a clear concentration-dependent response of the biosensor signal (Figure 2). Its magnitude and shape differed both in the case of intact and oxidized GAPDH and also in the case of the same manipulations with the biosensor flow cell containing immobilized actin, used as a control. This obviously reflects different modes of intact and oxidized GAPDH interaction with immobilized amyloid-β and actin. In order to elucidate affinity of these interactions we have determined *K*_d_ values for the complexes of intact and oxidized GAPDH with immobilized amyloid-β and actin.

**Figure 3:**
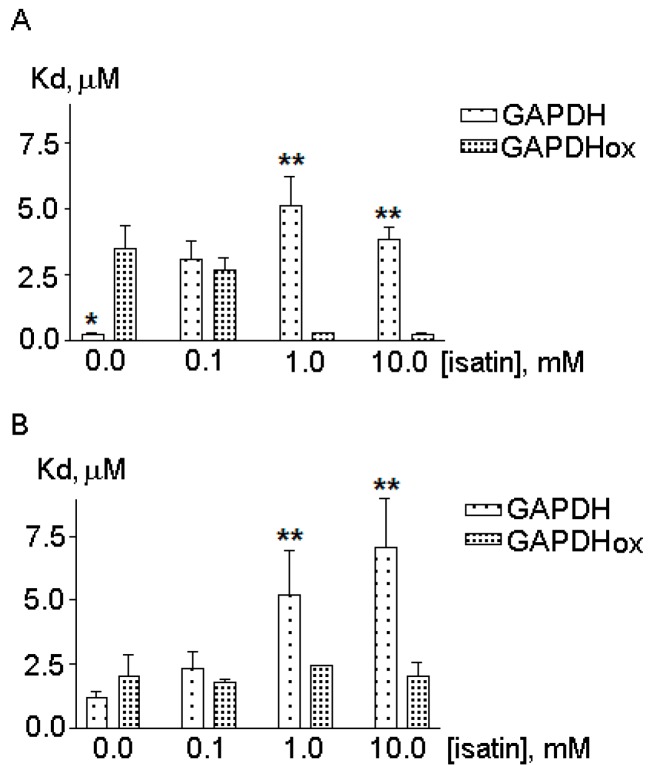
The effect of GAPDH oxidation on *K*_d_ values for GAPDH interaction with immobilized actin (**A**) and amyloid-beta (**B**). Data represent mean ± SEM from 3–5 independent experiments. Asterisks show statistically significant differences in *K*_d_ values for intact and oxidized GAPDH (* *p <* 0.05; ** *p <* 0.01) evaluated by paired Student’s *t*-test.

The calculated *K*_d_ value for the GAPDH amyloid-β complex of 0.22 ± 0.02 µM was in the range of *K*_d_ values for GAPDH complexes with actin reported in the literature [54]. In our experimental conditions GAPDH affinity to amyloid beta was even higher than to actin (the *K*_d_ value of 1.22 ± 0.2 µM, *p <* 0.01 *versus* the *K*_d_ value for GAPDH binding to amyloid-β) (Figure 2). Addition of the GAPDH cofactor, NAD (oxidized nicotineamide adenine dinucleotide) (0.01–5 mM), did not influence the behavior of the biosensor response implying lack of any influence of NAD (at least at physiological concentrations) on GAPDH binding to amyloid-β (data not shown), while 0.1 mM isatin decreased this binding and increased the *K*_d_ values (Figure 3).

In accordance with previous studies [54], GAPDH oxidation with 70 µM H_2_O_2_ was accompanied by a dramatic (more than 15-fold) decrease in catalytic activity of this enzyme (from 111 to 6 µmol/min per mg of protein) and also by a dramatic (more than 15-fold) increase in the *K*_d_ value for the enzyme interaction with amyloid-β (Figure 3A). This effect was specific for GAPDH interaction with amyloid-β as neither oxidation nor isatin significantly influenced GAPDH interaction with actin, one of the major cytoskeleton components (Figure 3B). This suggests that oxidative stress and isatin could influence profiles of amyloid-binding proteins in the brain.

### 2.3. Effects of Hydrogen Peroxide and Isatin on Proteomic Profiles of Rat Brain Amyloid-Binding Proteins

Indeed, the presence of 0.1 mM isatin significantly decreased the number of proteins specifically bound to amyloid-β to 28 (Table S2); however, only half of them were common with the group of amyloid-binding proteins of intact brain homogenate.

Induction of oxidative stress by incubating rat brain homogenates with hydrogen peroxide significantly influenced both the total number of amyloid-binding proteins specifically bound to the affinity sorbent and the profile of identified individual proteins (Table S3). The total number of identified proteins slightly decreased (*n =* 70) and comparison of the proteomic profiles of control and hydrogen peroxide treated brain homogenates revealed 50 common proteins.

**Figure 4:**
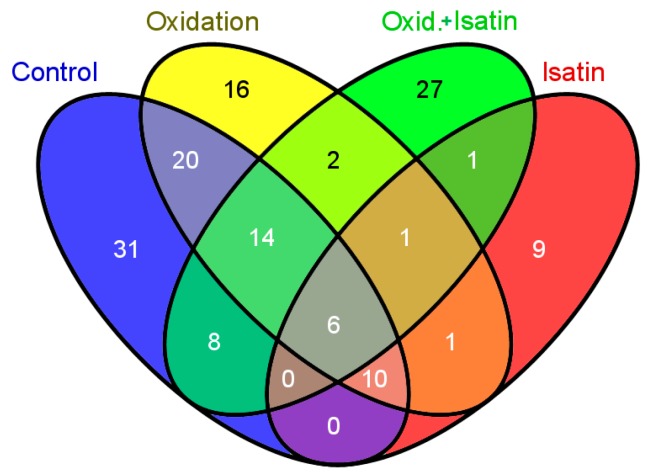
A Venn diagram illustrating the groups comprising the total number of coincided amyloid-binding proteins: control, oxidation, isatin, oxidation in the presence of isatin. Numbers of coincided proteins are shown within intersection areas and numbers outside intersections show proteins specific for each group.

Incubation of rat brain homogenate with hydrogen peroxide and isatin caused a further decrease in the total number of brain proteins bound to amyloid-β as compared with hydrogen peroxide treated brain homogenate (59 *versus* 70) (Table S4). However, the number of proteins coincided in brain homogenates treated with hydrogen peroxide in the absence and in the presence of 0.1 mM isatin were just 23, which is significantly less as compared to control and hydrogen peroxide treated homogenates (Figure 4).

In this context, it should be noted that among four groups of rat brain preparations, only six proteins coincided (Table 2); and only two of them, stress-70 protein and endoplasmin, are known to be oxidized in neurodegenerative diseases in patients and/or corresponding experimental models in animals.

**Table 2:** Common amyloid-binding proteins for all experimental groups.

No.	Protein Name	Intracellular Localization *	Oxidatively Modified/Altered in AD Brain and Different AD Models **
1	Carbamoyl-phosphate synthase [ammonia], mitochondrial	M	No
2	Betaine-homocysteine *S*-methyltransferase 1	C	No
3	Fructose-bisphosphate aldolase B	C, L, N, ER, CM	No
4	Endoplasmin **	ER	Yes
5	Stress-70 protein, mitochondrial **	M	Yes
6	Keratin, type II cytoskeletal 73	C	No

* Designation of intracellular localization is the same as in other tables; ** See Table 1 for details and corresponding references.

Thus, it appears that the repertoire of amyloid-beta binding proteins is influenced not only by oxidative stress but also by the presence of low molecular weight substances, which are obviously involved in the regulation of amyloid-β interaction with various subcellular proteins.

## 3. Discussion

Results of the present study indicate that a large group of intracellular brain proteins can specifically interact with amyloid-β. They belong to different intracellular compartments and different functional groups (Tables S1–S4 and Figure 1).

(Patho)physiological importance of the amyloid-β interaction with some of the identified proteins has been already demonstrated by other authors. For example, catalase inactivation by binding to amyloid-β [17] may explain intracellular accumulation of hydrogen peroxide followed by subsequent development of oxidative stress described in numerous studies. Some cytosolic proteins identified in this study as the amyloid-β binding proteins demonstrate reduced solubility in a transgenic mouse model of Alzheimer-type amyloidosis [16].

More than 30% of amyloid-β binding proteins are oxidized in AD brain and different AD models identified (Table 1). One of these oxidatively-modified proteins is GAPDH. This protein attracts special interest due to its multiple roles related to neurodegeneration and AD [23,55,56,57,58].

Being a classical glycolytic enzyme, GAPDH also exhibits various (patho)biological activities (see for review [23,40]) and is a putative target for neuroprotective drugs [26,59,60]. This redox sensitive enzyme can interact with both cytoskeleton components (e.g., actin) [61,62] and aggregate-prone proteins (e.g., amyloid-β) involved in the development and progression of some neurodegenerative disorders such as AD [23,57]. Several studies have demonstrated localization of GAPDH in plaques [55,56]. Binding of this enzyme to the recombinant cytoplasmic domain of β-amyloid precursor protein [20] and immobilized amyloid-β as the affinity ligand [21,63] have shown direct binding of brain GAPDH to both fibrillar and nonfibrillar forms of amyloid-beta peptide **1**–**42**. Although GAPDH also bound to fibrillar crystalline (used as negative control) its coprecipitation with amyloid-β occurred more effectively [21]. Our results of surface plasmon resonance (SPR)-based studies indicate that GAPDH directly binds not only to immobilized amyloid-β but also to actin; however, it also exhibits higher affinity to amyloid-β than to actin. Since physiological concentrations of NAD (cofactor of GADPH) have no influence on GAPDH interaction with amyloid-β, it appears that this interaction does not depend on catalytic properties of this enzyme.

It should be noted that GAPDH represents a good model protein for analysis of interaction of intracellular brain proteins with amyloid-β and the effect of oxidative stress and pharmacologically-relevant compounds on this interaction.

Oxidation of purified GAPDH caused a dramatic decrease in its affinity to amyloid-β as evidenced by a more than 15-fold increase in the *K*_d_ value (Figure 3B) for the complex oxidized GADPH immobilized amyloid-β. GAPDH was identified among amyloid-binding proteins after pretreatment of brain homogenate with hydrogen peroxide but it was not detected in the case of intact homogenate pretreated with 0.1 mM isatin (Tables S1 and 3). In this context, it is reasonable to suggest that the *K*_d_ values ranging from 2.5 to 3.5 µM (Figure 3) represent the lowest affinity limit, where binding of (oxidized) brain homogenate GAPDH to immobilized amyloid-β was still possible but the presence of 0.1 mM isatin prevented this binding.

As proved by both optical biosensor experiments and proteomic profiling, the presence of 0.1 mM isatin significantly decreased affinity of highly purified GAPDH to amyloid-β immobilized (Figure 3B) on the surface of the biosensor chip and also prevented binding of brain homogenate GAPDH to the affinity sorbent with immobilized amyloid-β as the affinity ligand.

Thus, different interaction behaviors of intact and oxidized purified GAPDH towards amyloid-β immobilized on the biosensor chip is generally consistent with changes in the proteomic profiles of intact and oxidized brain homogenates and the influence of isatin on these protein-amyloid beta interactions.

Isatin is an endogenous indole widely distributed in mammalian brain, peripheral tissues and body fluids [24,25,27]. Its physiological concentrations in blood can exceed 1 µM [64,65]. Although basal tissue concentrations usually vary from 0.1 to 1 µM, in some tissues much higher levels of isatin (40–70 µM) may also occur [27]. It interacts with all known biological targets in a readily reversible manner [24,25,27,29] and demonstration of its *in vivo* interaction with certain biological targets requires employment of special methodological approaches (e.g., [66]). Some experimental evidence exists that isatin may influence interaction of isatin binding proteins with cytoskeletal structures, important for both manifestation and attenuation of neurodegenerative disorders [29].

Mechanism(s), by which isatin decreases the number of proteins bound to the amyloid-β affinity sorbent, still needs clarification. Our results of model experiments with amyloid-β immobilized on the optical biosensor cuvette and (oxidized) GAPDH suggest direct interaction between isatin and amyloid-binding proteins. It appears that isatin competes with amyloid-β for binding to protein targets. By analogy with hydrogen-peroxide induced changes in GAPDH sensitivity to isatin (Figure 2B,C) we suggest that oxidative stress influences protein affinity to isatin and therefore the repertoire of amyloid binding proteins.

Thus, results of our proteomic study indicate that not only oxidative stress *in vitro* but also such non-peptide endogenous compounds as isatin significantly influence the repertoire of brain amyloid-beta binding proteins. The latter suggests that the interaction of amyloid-beta with particular intracellular proteins may be regulated not only by amyloid beta accumulation in various intracellular organelles, and by their redox status, but also by concentrations of endogenous or exogenously administered compounds influencing amyloid-β protein interactions. In this context, it is especially interesting in the present study that in the presence of 0.1 mM isatin only 10% of brain proteins detected coincided with the proteins recently detected as the detergent-insoluble proteins identified in forebrains of the APPswe/PS1dE9 (line 85) transgenic mice [16]; in the absence of isatin the proportion of coincided proteins exceeded 30%.

The isatin core has been used in the design of various compounds acting as inhibitors of apoptosis, anticonvulsants, and anxiolytics *etc.* [25,67] and administration of isatin improves symptoms of experimental Parkinsonism [68,69]. Studies with [^3^H]isatin revealed high density of isatin binding in hippocampus, and cortex [28,70,71], *i.e.*, the brain regions affected by amyloid-β deposition in various models of AD. Thus, it appears that the identity of a significant proportion of isatin- and amyloid-binding proteins may have certain biomedical importance, and isatin itself, or its numerous analogues tested in many laboratories for different purposes [27,67,72,73], may be a useful tool for studies of the interaction of amyloid-β and its intracellular targets and possibly as pharmacologically relevant compounds attenuating progression of AD in various experimental models. In this context, it is especially interesting that some recently synthesized isatin-3-arylhydrazones act as rather effective inhibitors of amyloid-β aggregation in model systems [74], while some other isatin derivatives are effective inhibitors of transthyretin fibrillogenesis related to (senile) amyloidosis [75].

**Figure 5:**
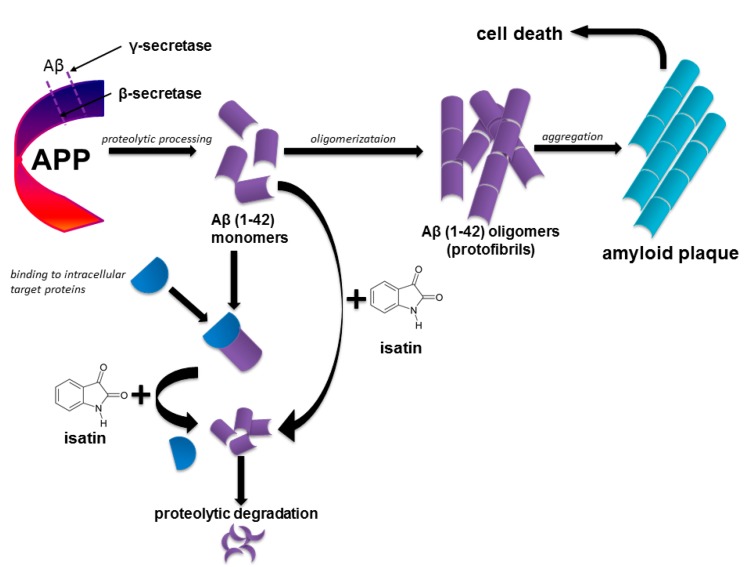
Proposed mechanism of isatin action in prevention of early molecular events leading to development of Alzheimer’s disease.

Taking into consideration data on the inhibition of aggregation of amyloidogenic proteins by isatin-based compounds, it is reasonable to suggest that endogenous isatin may be an effective neuroprotector at the very beginning of AD. Preventing interaction of amyloid-β with crucial intracellular targets (e.g., catalase, GAPDH, α-enolase *etc.*) isatin promotes their physiological functioning, while maintaining amyloid-β in the monomeric form isatin favors its intracellular degradation (Figure 5).

The proposed scenario does not rule out other mechanisms involving direct amyloid-β interaction with its intracellular targets. By analogy with amyloid-beta induced inactivation of catalase [17], it is possible that amyloid-β binding to GAPDH may contribute to energy deficit and impairments in fast axonal transport associated with inhibition/inactivation of this enzyme [76].

## 4. Experimental Section

### 4.1. Materials

Acetic acid (sodium salt), boric acid, formic acid, sodium tetraborate, and sodium hydroxide were from Acros Organics (Geel, Belgium). Affi-Gel 10 was purchased from BioRad (Hercules, CA, USA).

Reagents for the Biacore biosensor were obtained from GE Healthcare (Piscataway, NJ, USA). These included HBS-EP buffer (150 mM NaCl, 3 mM EDTA, 0.005% surfactant P20, 10 mM HEPES, pH 7.4); 10 mM acetate buffer, pH 4.0; amine coupling reagents kit, containing 1-ethyl-3-(3-dimethylaminopropyl) carbodiimide hydrochloride (EDC), *N*-hydroxysuccinimide (NHS) and 1 M ethanolamine–HCl, pH 8.5.

All other materials and chemicals including the human amyloid-β protein fragment (**1**–**42**) were from Sigma-Aldrich (St. Louis, MO, USA).

Rabbit muscle GAPDH was purified by the method of Scopes and Stoter [77]. The resulting enzyme preparations (specific activity 110–170 μmol/min per mg of protein as evaluated in the reaction of 3-phosphoglycerate reduction by NADH (reduced nicotinamide adenine dinucleotide) showed one band during electrophoresis in the Laemmli system. Before use, the purified enzyme was kept as an ammonium sulfate suspension at +4 °C for not more than two weeks.

### 4.2. Animals, Brain Homogenate Preparation and Incubations

Male albino rats (*n =* 24, 200–250 g) obtained from the Stolbovaya nursery (Russian Academy of Medical Sciences) were used in the experiments performed at least one week after their arrival from the nursery. Animals received a standard laboratory chow and water *ad libitum* and their decapitation was performed between 11.00 and 13.00 h under light ether anesthesia. All procedures conform to the Russian version of the Guide for the Care and Use of Laboratory Animals (Washington, 1996) and have been approved by the Animal Care and Use Committee of the Orekhovich Institute of Biomedical Chemistry issued on 16 June 2014 (identification number: EC3318-2014.2-IBMC).

The brains were immediately dissected and homogenized at a low speed in 0.05 M potassium-phosphate buffer, pH 7.4 (1:3, *w*/*v*), using an Ultra-Turrax T 10 homogenizer as described in [28,29]. In each experiment, the brain homogenates from 2 rats were aliquoted and incubated with 70 µM H_2_O_2_ in the absence or in the presence of 0.1 mM isatin at 37 °C for 30 min. Control brain homogenates also prepared from 2 rats were incubated during the same period without oxidation in the presence or in the absence of added isatin.

The mild oxidation of GAPDH was performed as described in [54] by incubating GAPDH with 70 µM H_2_O_2_ for 30 min at 20 °C in 50 mM phosphate buffer, pH 7.4.

### 4.3. Immobilization of Amyloid-β Protein Fragment **1**–**42** on Affi-Gel 10

Amyloid-β protein fragment **1**–**42** (1 mg) dissolved in 250 µL of distilled water was diluted with equal volume of 0.1 M Na-acetate buffer, pH 4.0. In parallel, an Affi-Gel 10 resin (0.5 mL) was washed on a glass filter with 10 volumes of water and 2 volumes of the same buffer. The resin was transferred to the 1.5 mL Eppendorf tubes and the buffer was eliminated by aspiration after centrifugation of resin slurry at 4000 rpm for 3 min using an Eppendorf centrifuge 5415R (Germany). Amyloid-β protein fragment was added to the mixture (1:1) and incubated at 4 °C overnight at a gentle stirring. The unbound protein fragment was removed by repeated centrifugations (4–5 times) as above in 1 M Tris-acetate buffer, pH 7.0. After addition of the same buffer the resultant suspension (1:1, *v*/*v*) was incubated at 4 °C for 4 h at a gentle stirring. After washing 4–5 times and resuspending in 50 mM potassium phosphate buffer, pH 7.4, containing 0.02% NaN_3,_ the Affi-Gel 10 resin with the immobilized amyloid-β protein fragment was stored at 4 °C.

All the above-mentioned incubations were performed with the control Affi-Gel, except the addition of amyloid-β protein fragment.

### 4.4. Affinity Interactions

After incubation at various conditions (see above) homogenates were lysed with 3% Triton X-100 (final concentration) at 4 °C for 1 h, diluted three-fold with 50 mM potassium phosphate buffer, pH 7.4, and centrifuged at 16,000 rpm for 20 min at 4 °C. The resultant supernatant (5 mg of protein/mL) was added to the suspension of the affinity sorbent (Affi-Gel 10 with the immobilized amyloid-β protein) (1:1 *v*/*v*). Bacitracin, aprotinin, and PMSF were added up to the concentrations of 0.005%, 0.01%, and 1 mM, respectively, and the suspension was incubated overnight at 4 °C at a gentle stirring. Such incubation was previously used for the analysis of protein interactions with the recombinant cytoplasmic domain of amyloid-β precursor protein [20]. The same incubations were also performed with the control Affi-Gel 10 without immobilized protein ligands.

After the incubation the sorbent was washed with the same potassium phosphate buffer up to the absence of the protein in the washing (evaluated by baseline at D_280_) and then packed into the columns (1 × 0.75 mL). Proteins were eluted from the column by 1 M glycine buffer, pH 2.8, containing 150 mM NaCl at a flow rate of 0.5 mL/min. The eluate was concentrated up to 0.200 mL using Amicon Ultra centrifugal filter devices (Millipore, Hessen, Germany) [29]. Proteins were extracted, modified, and digested on filters using the recently published FASP (filter-aided sample preparation) protocol [78].

The peptide samples were analyzed using Ultimate 3000 nano-flow HPLC (Thermo Scientific, Waltham, MA, USA) connected to a LTQ Orbitrap Elite (Thermo Scientific, Waltham, MA USA) mass spectrometer equipped with a Nanospray Flex NG ion source (Thermo Scientific, Waltham, MA USA). Peptide separation was carried out on a RP-HPLC column Zorbax 300SB-C18 (C18 particle size of 3.5 µm, inner diameter of 75 µm and length of 150 mm) using a linear gradient from 95% solvent A (water, 0.1% formic acid) and 5% solvent B (water, 0.1% formic acid, and 80% acetonitrile) to 60% solvent B over 85 min at a flow rate of 0.3 µL/min.

Mass spectra were acquired in the positive ion mode. Data was acquired in the Orbitrap analyzer (Thermo Scientific, Waltham, MA USA) with resolution of 60,000 (*m*/*z* 400) for MS and 15,000 (*m*/*z* 400) for MS/MS scans.

Survey MS scan was followed by MS/MS spectra of the five most abundant precursors. For peptide fragmentation higher energy collisional dissociation (HCD) was used; the signal threshold was set to 10,000 for an isolation window of 2 *m*/*z* and the first mass of HCD spectra was set to 100 *m*/*z*. The collision energy was set to 35 eV. Fragmented precursors were dynamically excluded from targeting for 60 s. Singly charged ions and ions with undefined charge states were excluded from triggering MS/MS scans.

Protein identification was performed using MASCOT software (www.matrixscience.com). All tandem mass spectra were searched against the SwissProt database (release 2014_02, [79]) The following search parameters were used: Trypsin was used as the cutting enzyme, mass tolerance for the monoisotopic peptide window was set to ±20 ppm, the MS/MS tolerance window was set to ±0.05 Da and one missed cleavage was allowed. Cysteine carbamidomethyl and oxidized methionine were chosen as variable modifications. The criteria of positive identification were set as following: Minimum score of 50, at least three positive identifications from three different runs.

### 4.5. SPR (Surface Plasmon Resonance) Measurements

GAPDH interaction with immobilized amyloid-beta and actin has been studied using an optical biosensor Biacore 3000 (GE Healthcare, Piscataway, NJ, USA) and the Biacore Control software (GE Healthcare) for instrument operation. All the experiments were performed with the standard optical chips Biacore CM5. Molecular interactions were registered as sensograms representing the records of biosensor signals in resonant units (RU) as time function. The interactions were estimated by subtracting the biosensor signal of a blank flow cell from the signal of the cell with the immobilized ligand or protein.

Covalent immobilization of the proteins (Figure 1) on the sensor chip included surface activation by the 0.2 М EDC/0.05 М NHS mixture and subsequent injection of the solutions (100 µg/mL) of actin or amyloid-β in 10 mM acetate buffer, pH 4.0, at a flow rate of 5 µL/min for 10 and 30 min, respectively. Washing and residual active group inactivations were performed as described in [29].

Protein–protein interactions were monitored by injecting at least five different concentrations of native or oxidized GAPDH (1–10 μM) dissolved in 50 mM potassium phosphate buffer, pH 7.4 (running buffer), at a flow rate of 5 μL/min for 5 min. Each experiment was repeated from three to five times using three different CM5 cuvettes with three different commercial preparations of immobilized amyloid-β peptide. The sensor surface was regenerated after each measurement cycle by washing with 1 M NaCl in the running buffer and 50 mM glycine, pH 9.5, for 0.5 min at a flow rate of 50 μL/min.

Data analysis was performed by applying “Biacore Evaluation v. 4.1” software. To determine the equilibrium dissociation constant (*K*_d_ = 1/*K*_a_) the equilibrium data were fitted to a single-site model (Equation (1)):

(1) $$R_{\text{eq}}=(R_{\text{max}}\cdot C\cdot K_{a})/(1+C\cdot K_{a})$$

where *R*_max_ stands for the maximal response; *C* is the concentration of a protein; *R*_eq_ is the equilibrium response at a given protein concentration; and *K*_a_ is the equilibrium association constant.

### 4.6. Statistical Analysis

Statistical significance of differences was evaluated by paired Student’s *t*-test. Differences were considered as statistically significant at *p <* 0.05.

## 5. Conclusions

In this study we have investigated profiles of amyloid-binding proteins isolated from control rat brain homogenates and rat brain homogenates treated with hydrogen peroxide (oxidative stress simulation), the small non-peptide endogenous regulator isatin (indole-2,3-dione), and homogenates treated with hydrogen peroxide in the presence of isatin. Proteomic profiling of control rat brain homogenates resulted in identification of 89 individual intracellular amyloid-binding proteins and approximately 25% of them were previously identified as isatin-binding proteins. More than 30% of the amyloid-binding proteins are differentially expressed or altered/oxidatively modified in AD patients. Incubation of brain homogenates with 70 µM hydrogen peroxide significantly influenced the profile of amyloid-β binding proteins and 0.1 mM isatin decreased the number of identified amyloid-β binding proteins both in control and hydrogen peroxide treated brain homogenates. The effects of hydrogen peroxide and isatin have been confirmed in optical biosensor experiments with purified glyceraldehyde-3-phosphate dehydrogenase, one of the known amyloid-β binding proteins also identified in this study. Our results suggest that isatin protects crucial intracellular protein targets against amyloid binding. This effect possibly favors intracellular degradation of this protein via preventing formation of amyloid-β oligomers described in the literature for some isatin derivatives. If such a scenario occurs *in vivo*, it appears that endogenous and/or pharmacological compounds decreasing amyloid-β interaction with numerous amyloid-binding proteins in the brain would be especially effective at early stages of AD, when toxic amyloid-β oligomers have not yet been formed.

## Supplementary Materials

Supplementary tables can be found at http://www.mdpi.com/1422-0067/16/01/0476/s1.
